# Music as an ergogenic aid in team sports: a systematic review

**DOI:** 10.3389/fspor.2025.1514756

**Published:** 2025-05-13

**Authors:** Eduard Bezuglov, Timur Vakhidov, Elizaveta Kapralova, Georgiy Malyakin, Mikhail Vinogradov, Sergey Chyogin, Mikhail Butovskiy

**Affiliations:** ^1^ Department of Sports Medicine and Medical Rehabilitation, I.M. Sechenov First Moscow State Medical University, Ministry of Health of the Russia Federation, Moscow, Russia; ^2^ Federal Scientific and Clinical Center for Sports Medicine of the Federal Biomedical Agency, Moscow, Russia; ^3^ Department of Rehabilitation and Sports Medicine, Kazan State Medical University, Kazan, Russia

**Keywords:** ergogenic aids, loads, performance, load tolerance monitoring, music

## Abstract

**Objectives:**

Enhancing physical performance and improving load tolerance through safe methods is a priority for most athletes. One potentially beneficial approach is listening to music, which exerts positive effects through various mechanisms. This study aims to investigate the influence of music on athletic performance and endurance, focusing specifically on its potential as an ergogenic aid in team sports—an area that has received less attention compared to individual sports.

**Methods:**

To examine the effects of music on physical performance and load tolerance in team sports athletes, we conducted a systematic search for original English-language articles in PubMed, Mendeley, and the Cochrane Library from inception to June 2024, following PRISMA guidelines.

**Results:**

The search identified eight studies that met the inclusion criteria, involving 140 participants from football, volleyball, and basketball. All studies demonstrated a low risk of bias. None of the studies included elite adult international-level athletes. The analysed parameters included peak power, sprint and jump performance, maximal oxygen consumption, repeated sprint ability, change of direction, and load tolerance indicators such as heart rate, rating of perceived exertion (RPE), and fatigue index. Most studies demonstrated a significant positive effect of music on these parameters; however, the protocols for music accompaniment were not standardised.

**Conclusion:**

The findings suggest that music can positively impact both physiological and psychological factors, though its application in team settings requires further investigation. Given its safety and accessibility, music may serve as a valuable tool for enhancing performance in various sporting contexts. Future studies should include more detailed information on music usage protocols and involve larger sample sizes, particularly including adult elite athletes.

## Introduction

Enhancing performance is a key priority for professional athletes. It can be improved by directly enhancing various physical parameters and by improving load tolerance and accelerating post-load recovery (1, 2). Effective ergogenic aids must be well-tolerated, safe, and compliant with anti-doping regulations (3, 4). At the highest competitive level, success is often determined by fractions of a second, and most competitors regularly use permitted dietary supplements and substances to improve performance (5–7). However, the list of permitted substances with proven efficacy is limited (8).

In this context, non-pharmacological aids that boost performance without risking anti-doping violations are gaining momentum. These include mouth rinses (9), music listening (10), body cooling during competitions in high temperatures (11), transcranial brain cortex stimulation (12), and other methods.

It is not surprising that the number of studies in this area is increasing. One of the most studied methods for enhancing performance is music listening, whose influence on various physical indicators began to be investigated last century (13, 14), but has become more widespread in the last decade (10). Regarding sports performance, music listening has been shown to increase anaerobic performance, improve perceptual-cognitive skills, and reduce rate of perceived exertion (RPE) (15, 16).

Possible mechanisms for enhancing performance through music are thoroughly described in reviews by Ballman and Terry et al. These mechanisms involve a combination of psychological, physiological, and psychophysiological factors (17, 18). Listening to music can enhance mood, reduce perceived fatigue, and increase positive emotions (19). For example, Hutchinson et al. found that participants who listened to music while running could maintain high intensity for longer while perceiving less exertion (20). Additionally, music during warm-up can increase alertness and peak power in Wingate tests among elite sprinters (21).

Music primarily affects physiological responses in the nervous and metabolic systems. Bigliassi et al. found that performing motor tasks while listening to music increased activity in the left inferior frontal gyrus (22). Activation of this brain region is associated with reduced RPE and improved movement organization. According to Jia et al., music listening during cycling can enhance parasympathetic reactivation and reduce heart rate variability after exercise (23). Music listening during physical exercise can also increase oxygen consumption and cardiac output, improving muscle oxygenation and reducing blood lactate levels (24–26). During physical activity, psychophysiological changes like increased arousal, reduced RPE, and improved autonomic control are observed. These effects are believed to stem from the combined influence of music on both psychological and physiological processes (18).

The results of the most recent systematic review and meta-analysis examining the effects of pre-task music on exercise performance and associated psychophysiological responses show that pre-task music enhances psychological responses and alleviates fatigue-related symptoms associated with exercise performance improvement. This effect is particularly pronounced when self-selected music is used and is more evident in active individuals than in trained athletes (10). However, the included studies mainly focused on healthy populations not involved in professional sport and were limited to listening to music exclusively before physical activity. In particular, despite evidence of the beneficial effects of music on various performance parameters, such as peak and mean power (27), repetitive sprint ability (28), speed (29), endurance (30), and mitigation of heat-related declines in exercise performance (31). These studies involve non-elite athletes and amateurs from individual sports, lack high-intensity physical testing to failure, and do not include sport-specific skill testing. Most existing studies have several limitations that hinder the extrapolation of their findings to team sports, particularly at the professional athlete level.

Another critical aspect to consider is the inapplicability of protocols used in individual sports to team-based settings. One of the key factors determining the effectiveness of music as an ergogenic aid is individual preference (18). It is almost impossible to select a single type of music that would be universally preferred by all team members. Consequently, in team sports, listening to music as an ergogenic strategy is likely to be most effective during training sessions where individual listening devices (e.g., portable headphones) can be used. However, this approach also presents challenges due to the need to maintain communication between team members. Thus, in team sports, music can primarily be used as an ergogenic aid during training or pre-competition through individual listening devices such as portable headphones.

Given the limitations of previous work, it may be of practical relevance to investigate the effects of different music application protocols as a potential ergogenic strategy in team sports. This systematic review aims to assess the ergogenic effects of music on physical performance and load tolerance in team sports athletes, providing a foundation for understanding its broader potential, including possible influences on sport-specific skills. This systematic review addresses the following question using the PICOS framework: In healthy professional team sports athletes (Population), does listening to music (Intervention) compared to no music (Comparison) enhance physical performance and load tolerance (Outcomes), as evaluated in randomized controlled trials (Study Design)?

## Materials and methods

### Search strategy

To identify studies on the effects of music listening on performance and load tolerance in healthy professional team sports athletes, a search was conducted in PubMed, Mendeley, and the Cochrane Library databases from their inception to June 16, 2024, according to PRISMA guidelines (32). The work within this study, including selection, analysis, and evaluation, was conducted from June to August 2024, with continuous updates based on newly released research findings. The search query was: (music OR melody OR “listening to music” OR “music playback” OR “music playing” OR “music listening” OR “musical intervention” OR “musical therapy” OR “background music” OR “music exposure” OR beats) AND (performance OR “technical performance” OR “physical performance” OR “mental performance” OR “physical indicators” OR “physical fitness” OR “loads” OR “workload” OR “recovery” OR “endurance” OR “stamina” OR “maximal aerobic power” OR “cardiorespiratory fitness” OR “strength” OR “muscular strength” OR “speed” OR “coordination” OR “ergogenic aids” OR “recovery state” OR “psychological recovery” OR “well-being” OR “mental state” OR “mood state”) AND (“football players” OR “soccer players” OR “footballers” OR “soccer athletes” OR “athletes” OR “sports players” OR “team sports”). No search filters or limits were applied; only the specified search query was used in each of the databases.

All relevant articles found were reviewed. The PICOS criteria were used to formulate the eligibility criteria (33):

- P (Population): Healthy athletes participating in team sports at various levels of sporting proficiency.
- I (Intervention): Listening to various types of music (different frequencies, through different devices).
- C (Comparison): Comparison was made between participants who listened to music during the experiment and those who did not.
- O (Outcome): Various changes in performance, load tolerance, and fatigue levels were analysed.
- S (Study Design): The review included original randomized controlled trials with parallel groups or crossover design conducted on humans.

### Study selection

The inclusion criteria for this review were as follows: studies published in English, original randomized controlled trials with parallel groups or crossover design conducted on humans, and studies examining the effects of listening to various types of music (different frequencies, through different devices). No specific exclusion criteria were applied beyond these parameters.

An initial literature search created a selection of studies covering various aspects of music listening. The studies were analysed based on design, participant characteristics, performance assessment methods, musical conditions, experiment duration, and other relevant factors. The studies were classified by type of sport and experimental results. After analysing the publications, the results were compared, and conclusions were drawn about the effects of music listening on physical performance and load tolerance in healthy professional team sports athletes. These studies were analysed in detail by two independent researchers, V.T.M. and K.E.S. In cases of disagreement on data interpretation, a senior expert, B.E.N., was consulted. The search results were downloaded and filtered in the Mendeley Reference Manager v2.64.0 (Mendeley Ltd. UK) systematic review software. A manual search helped identify other suitable articles in eligible full-text articles to be incorporated in the systematic review. A consensus was formed on the final studies included. All identified studies were assessed for risk of bias employing the revised Cochrane Risk of Bias Tool for Randomised Trials (RoB 2) (34). The RoB2 was covering the following evaluation domains: bias arising from the randomization process, bias due to deviations from intended interventions, bias due to missing outcome data, bias in measurement of the outcome and bias in selection of the reported result. Two independent reviewers V.T.M. and K.E.S assessed the risk of bias, and in cases of disagreement, a third reviewer B.E.N. was consulted to reach a consensus. This approach ensures the reliability and reproducibility of our findings.

This systematic review was submitted for registration in PROSPERO; however, the registration was declined as PROSPERO does not accept systematic reviews related to performance in sports. To ensure transparency, we adhered to rigorous methodological guidelines and provided a detailed description of our review protocol within this manuscript.

### Synthesis of result

Due to significant variability in music protocols (e.g., selection criteria, tempo ranging from 60 to >140 bpm, and listening methods such as individual headphones or shared stereo systems), a narrative synthesis was chosen over a meta-analysis to qualitatively summarize the findings.

## Results

### Study selection

**Figure 1:**
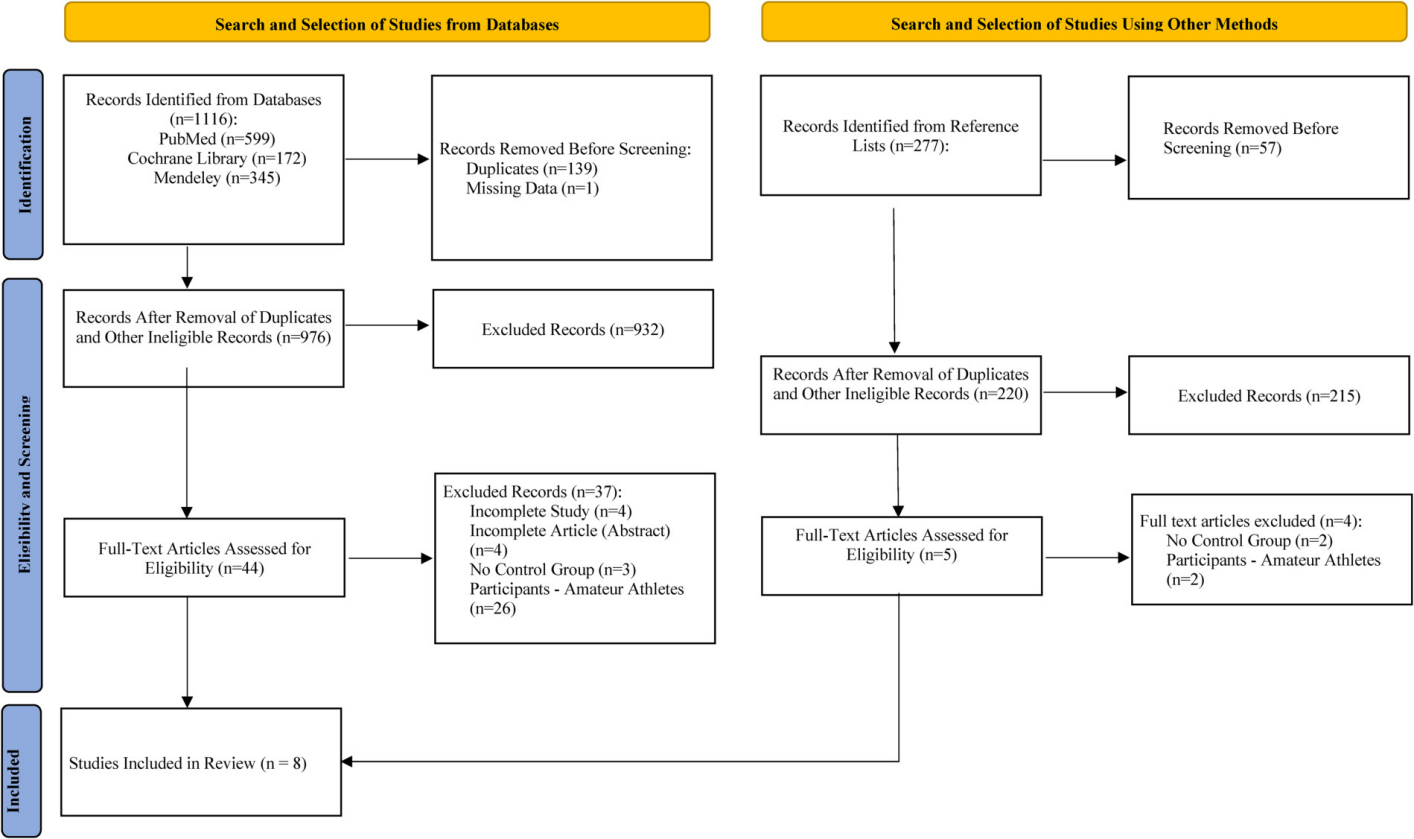
Process of study selection.

Based on keywords and their combinations, 1,116 records were found in the analysed databases. Subsequently, 140 records were excluded (139 duplicates, and 1 article was removed due to lack of data). After analysing titles and abstracts, 932 records were excluded because they did not meet the main criteria and topic of this systematic review. Of the remaining 44 studies, 38 were excluded after thorough screening: 4 had no research results (incomplete study), 4 were abstracts only, 3 lacked a control group, and 27 involved amateur athletes. Additionally, reference lists of each study were reviewed to minimize the risk of missing suitable studies for this systematic review. Out of 277 documents in reference lists, 2 studies met all PICOS criteria and were included in the review. Finally, 8 publications in English describing studies with healthy professional team sports athletes investigating the effects of music listening on performance and load tolerance were identified (Figure 1, Table 1).

**Table 1:** Studies on the effects of music listening on various indicators in healthy professional team sports athletes.

Title, year, authors	Design	Number and level of participants	Music selection	Music tempo	Music volume	Listening equipment	Music protocol	Study protocol	Control	Analysed parameters	Results
Gavanda et al., (41)	Crossover	13 male volleyball players (1st-3rd division) Age: 25.5 ± 2.63 years; Height: 187.3 ± 7.47 cm; Weight: 83.9 ± 8.36 kg	By participants/researchers	PS—124.7 ± 19.4 bpm PI—76 bpm	Not specified	Noise-cancelling headphones + MP3 player (even when no music was playing)	For 1 min before testing + during testing (no more than 2 min). Testing began 1 min after music started.	Mood questionnaire—warm-up—music listening—5 SJ (rest time between jumps determined by participants)—mood questionnaire	No music	Height and power of SJ; emotions and mood after testing.	PS and PI: SJ height and power =; Rest time =; Emotions, mood =
Belkhir et al., (35)	Crossover	12 male footballers (2nd division) Age: 21.82 ± 2.47 years; Height: 1.78 ± 0.04 m; Weight: 71.26 ± 4.24 kg	By participants/researchers	PS—120–140 bpm PI—120–140 bpm	Not specified	Personal headphones (even when no music was playing)	During warm-up Duration: 10 min	Warm-up—testing (30-sec shuttle sprint×6, rest between sets 35 s)	No music	RPE after warm-up and test; well-being after warm-up and test; Total distance (TD), Maximum distance (MD), and Fatigue index (FI) during test	PI: TD↑; MD↑; PS: TD↑; MD↑; Well-being after test↑; RPE after test↑
Belkhir et al., (36)	Crossover	15 male footballers (2nd division) Age: 21.02 ± 1.52 years; Height: 1.76 ± 0.06 m; Weight: 74.43 ± 9.13 kg	By participants/researchers	PS—120-140 bpm PI—60 bpm	Standard volume	Personal headphones (even when no music was playing)	During warm-up Duration: 10 min	Warm-up—mood assessment (scale)—testing—30-sec continuous SJ	No music	Body temperature, Max height (MH), Avg height (AH), FI, Well-being	PI: MH↑; AH↑; FI↓; Well-being↑; PS (compared to control and PI): Body temperature =; MH↑; AH↑; FI↓; Well-being↑
Blasco-Lafarga et al., (38)	Crossover	13 male basketball players (national U-16 team) Age: 14.85 ± 0.68 years; Height: 1.88 ± 0.05 m; Weight: 75.89 ± 8.02 kg	Researchers compiled song list, participants chose from it	LMM—113.91 ± 30.68 bpm HMM—116.02 ± 17.30 bpm	Not specified	Not specified	During testing Exact listening duration not provided	Warm-up—5 min rest—measure indicators (HR, PRR, SaO2, blood lactate)—testing (30-15IFT and V-cut tests)—5 min rest—measure indicators (HR, PRR, SaO2, blood lactate, RPE, VO2max)	No music	30-15IFT: Before: HR, PRR, SaO2, blood lactate; VO2max; After: HR, PRR, SaO2, blood lactate, RPE; V-cut test: Before: HR, PRR, SaO2; After: HR, PRR, SaO2, RPE	HMM (compared to control and LMM): ↑30-15IFT; V-cut test =; VO2max↑; HR =; SaO2 =; Blood lactate =; RPE = LMM: 30-15IFT =; V-cut test =; VO2max =; HR =; SaO2 =; Blood lactate =; RPE↓ (control + HMM) (easier)
Hammami et al., (40)	Crossover	18 male basketball players (national team) Age: 17.2 ± 0.6 years; Height: 189.8 ± 7.0 cm; Weight: 71.6 ± 6.7 kg	By participants	PS –>120 bpm	Comfortable and moderate	Headphones (MP3 player), (even when no music was playing)	During testing Playlist duration: 10 min, exact listening duration not provided	Warm-up—10 sprints (R-CoD)—30 s rest between attempts	No music	Total time (TT); Peak time (PT); FI	TT↑; PT↑; FI =
Eliakim et al., (42)	Crossover	24 volleyball players—12 men, 12 women (national team) Men: Age: 17.0 ± 0.2 years; Height: 189.3 ± 1.2 cm; Weight: 78.7 ± 1.8 kg; Women: Age: 16.4 ± 0.3 years; Height: 172.3 ± 1.4 cm; Weight: 66.6 ± 1.5 kg	By researchers	140 bpm	70% of maximum	Stereo CD player	During warm-up Playlist duration: 11 min 39 s, exact listening duration not provided	Warm-up—testing without additional rest (Wingate test)	No music	Average HR during warm-up; RPE after warm-up and test; Peak power (PP), Average power (AP); FI	Average HR during warm-up↑; RPE after warm-up↑; RPE after test =; PP↑; AP =; FI =
Tounsi et al., (37)	Crossover	33 footballers—19 men, 14 women (professional league) Men: Age: 17 ± 0.3 years; Women: Age: 17 ± 0.2 years	By researchers	>130-140 bpm	Not specified	Headphones (even when no music was playing)	During warm-up Duration: 15 min	Warm-up—RSA testing (20 m)	No music	RPE after warm-up and RSA; Affective workload assessment (AWA); Best sprint time (RSAb); Mean sprint time (RSAm); Sprint decrement % (RSAd%)	Men: AWA after warm-up↑; RSAb =; RSAm =; RSAd% = Women: AWA after warm-up=; RSAb =; RSAm↑; RSAd%=
Eliakim et al., (39)	Crossover	12 male basketball players (first division Israeli youth league) Age: 16 ± 0.5 years; Height: 185.9 ± 0.9 cm; Weight: 75.5 ± 6.1 kg	By researchers	>120 bpm	70 decibels	Stereo CD player	During testing Playlist duration: 11 min, exact listening duration not provided	RSA testing 12 × 20 m	No music	RPE before and after RSA test; RSAb; RSAm; RSAd%; RSAt; RSA1-12; Average HR during test; HR at end of test	Average HR = ↑; RSA11 = ↑; RSA12 = ↑; RSA1-10 =; RSAb =; RSAm =; RSAd% =; RSAt =; HR at end of test =

VO2max, maximal oxygen consumption; RPE, rating of perceived exertion; PRR, Perceived readiness; HR, Heart rate; PP, Peak power; AP, Average power; FI, Fatigue index; PT, Peak time; MH, Maximum height; AH, Average height; TD, Total distance; MD, Maximum distance; SaO2, Blood oxygen saturation; bpm, Beats per minute; BMI, Body mass index; TT, *T*-test; RSA, Repeated sprint ability; R-CoD, Repeated change of direction sprints; IFT, Intermittent fitness test; PS, Participant-selected music; PI, Investigator-selected music; LMM, Low-motivation music; HMM, High-motivation music; TT, Total time; ↑, Increase in indicators; ↓, Decrease in indicators; =, No effect; RSAb, Best sprint time; RSAm, Mean sprint time; RSAd%, Sprint decrement percentage; RSAt, Total sprint time; RSA1-12, Time for corresponding sprint; AWA, Affective workload assessment; 30-15IFT, Intermittent fitness test to failure, including maximum possible rounds of 30-second running, separated by 15-second rest; V-cut test, 4 sprints of 5 meters each with a change of direction in each sprint.

### Study participants

The total number of research participants was 140, comprising 114 males and 26 females—athletes from team sports—with an average age ranging from 14.85 to 25.5 years. In three studies, the participants were football players whose level of sports training ranged from semi-professional (35, 36) to elite athletes (37). A further three studies involved elite basketball players (38–40). Two studies focused on volleyball players from the 1st to 3rd divisions (41) and the national team (42).

### Music protocol

In three studies, the choice of music played was made by the researchers (37, 39, 42). In two studies, the music was chosen by the participants (38, 40), whereby in one of these studies the participants had to choose music from a list provided by the researchers (38). In the remaining studies, the music was selected by both the participants and the researchers, with the groups split accordingly (35, 36, 41).

The tempo of the selected music was up to 120 bpm (35, 41) and higher in all studies, including (35, 41). In the study by Blasco-Lafarga et al., the tempo ranged from 'slow' to “fast” music (113.91 ± 30.68 bpm and 116.02 ± 17.30 bpm, respectively), which participants classified as low and high motivating music (38).

Only one study reported an exact value for volume (70 decibels) (39)]. Another study mentioned that the sound intensity was set at 70% of the maximum possible level (42). Two other studies indicated that the intensity was moderate and comfortable (36, 40), while the remaining studies did not provide descriptions of the sound intensity (35, 37, 38, 41). Five studies used individual headphones (35–37, 40, 41), two used a common stereo system (39, 42), and one did not specify (38). The duration of music playback did not exceed 2 min (41), 10 min (35, 36) or 15 min (37). The remaining studies did not report the exact duration of listening. However, Eliakim et al., 2012, Eliakim et al., 2007 and Hammami et al., 2021 reported the total duration of the music playlist (39, 40, 42).

### Outcomes

The results are presented in Table 1 and included jump test height and power, sprint test distance and time (e.g., RSA, R-COD, V-cut test, 30-15 IFT) and power measured during the Wingate anaerobic test. In addition, psychometric indicators, RPE, heart rate during different phases of physical activity, lactate level, VO2max, SaO2 and body temperature were analysed.

### Risk of bias/quality appraisal

Most studies included in this review can be considered high methodological quality: all were randomized with sufficient sample size and control groups (Table 2). The studies included in this review conducted their own sample size calculations using various established methodologies. Each study justified its sample size based on statistical considerations, such as power analysis, effect size estimation, or adherence to previously validated study designs. As a result, the included studies reported sufficient sample sizes to support the validity of their findings, which collectively strengthen the reliability of our review.

**Table 2:** Risk of bias in studies included in this systematic review.

Study	D1	D2	D3	D4	D5	Overall
Gavanda et al. (41)	+	+	+	+	+	+
Belkhir et al. (35)	?	+	+	+	+	+
Belkhir et al. (36)	?	+	+	+	+	+
Blasco-Lafarga et al. (38)	−	?	+	?	+	?
Hammami et al. (40)	+	+	+	+	+	+
Eliakim et al. (42)	?	+	+	+	+	+
Tounsi et al. (37)	+	+	+	−	−	+
Eliakim et al. (39)	+	+	+	+	+	+

**Domains: D1,** Bias arising from the randomisation process; **D2,** Bias due to deviations from intended interventions; **D3,** Bias due to missing outcome data; **D4,** Bias in measurement of the outcome; **D5,** Bias in selection of the reported result; “**–”,** Some concerns; “**+”,** Low concerns; “**?”,** Unclear risk of bias.

## Discussion

This review highlights the growing interest in music as a performance enhancer, with seven of the eight included studies published within the last five years. This may be due to existing data on the positive effects of music listening on performance and load tolerance in individual sports athletes (43, 44).

In accordance with the predefined criteria, the review included only studies involving athletes from team sports. Most frequently, the participants were young athletes, which is reasonable given that the use of various substances as ergogenic aids may be challenging in this cohort. The participants' level ranged from semi-professional to elite athletes; however, no elite adult athletes were included.

Music was most frequently listened during warm-up before testing in four studies included in this review (35–37, 42), which is likely the most replicable protocol under team sports conditions. In three studies, music was listened to during physical testing (38–40), and in one, music started one minute before testing and continued during testing (41).

Notably, no studies compared music listening during different periods of training (e.g., warm-up vs. sport-specific load or rest), although there is evidence that the effect of different tempo music may vary depending on the time of day (35, 36).

Additionally, the studies used varied music listening protocols. Music was presented both through personal headphones and through a stereo system. Individual headphones are likely more optimal as they allow athletes to choose their preferred music and change it at any time. According to Belkhir et al. and Bentouati et al., participant-chosen music had a greater impact compared to researcher-chosen music (35, 36, 45). These findings support Ballmann et al.'s review, noting that preferred music has the most significant effect on various performance aspects (18). It is worth noting that future studies should consider the convenience of using headphones during physical activity, as well as the impact of headphone use on physical performance. This is especially important if participants in the control group perform the same tasks while wearing headphones without sound, as the presence of a noise-canceling mode may trigger various physiological responses (46, 47).

Only one study provided precise digital values for sound intensity (70 decibels) (39). Another mentioned that intensity was 70% of the maximum possible (42), two described intensity as moderate and comfortable (36, 40), and the rest did not describe sound intensity (35, 37, 38, 41). The analysed studies did not compare the effects of music at different sound intensities; however, previous research involving healthy individuals, amateur athletes, and highly trained taekwondo athletes demonstrated significant benefits from using music with higher intensity/volume (48–50). Five studies limited the tempo to >120 bpm (35, 37, 39, 40, 42), while others varied the tempo from 60 to 140 bpm (36, 38, 41). Music with a tempo >120 bpm showed more positive effects on performance indicators compared to slower music (36, 38). A more detailed description of the music playback protocol would enhance comparability, reproducibility, and practical recommendations for athletes and their coaches.

All studies except one (41) provided evidence of the positive effects of music listening on various physical performance indicators (power, speed, jump height, VO2max) and load tolerance (heart rate, RPE, fatigue index).

One study found that low-motivation music significantly reduced RPE compared to high-motivation music and control group (38). In the high-motivation music group, there was a significant performance improvement in the 30-15 IFT test, which could explain the greater load in this group and, consequently, the absence of a significant decrease in RPE. Another study showed that RPE was significantly higher after testing (35) and warm-up (37, 42) compared to control groups, potentially due to increased performance and activity levels during these phases. Other studies did not include psychological factors such as RPE in their protocols; however, listening to music was shown to improve athletes' well-being after exertion (35, 36).

A significant reduction in the fatigue index was found in only one of three studies analysing this parameter (36). However, the absence of significant changes in the fatigue index in studies by Eliakim et al. and Hammami et al., with simultaneous significant increases in performance in anaerobic tests, likely indicates better load tolerance among participants listening to music (40, 42).

Regarding the physiological effects of music, two out of three studies found that listening to music significantly increased average heart rate during physical activity (39, 42), while post-exercise heart rate and recovery heart rate remained unchanged (42). One study also demonstrated a significant increase in VO_2_max, along with improved 30-15IFT test performance in the music group compared to the control group and low motivation music group (38). Other physiological parameters, including lactate levels, body temperature, and blood oxygen saturation, were assessed, but no significant changes were observed.

Regarding strength performance, data on the positive effects of music are contradictory. Belkhir et al. found significant increases in maximal and average jump height (36), whereas Gavanda et al. found no significant changes (41). Increased peak power during the Wingate test was observed, independent of gender, in both male and female participants (42). Tounsi et al. noted that listening to music during warm-up before the RSA test can improved performance in women, it only affects internal load in men (37). Most studies showed positive effects of music on sprint tests, including significant increases in sprint speed during the 30-15 IFT test, reduced sprint time during repeat sprint ability and repeat change of direction tests, and increased total and maximal distance in shuttle sprint tests (35, 37–40).

### Limitations

While this review demonstrates music's positive effects on general physical performance indicators, it does not extensively address sport-specific skills such as technical execution under pressure or decision-making in game-like scenarios. These skills are integral to team sports performance, and future studies should investigate how music influences them, potentially through protocols simulating competitive conditions.

A notable limitation of the included studies is the absence of elite international-level athletes. While the participants were professional athletes from football, volleyball, and basketball, their competitive level may not reflect the demands of top-tier international competition. Future studies should prioritize including elite athletes in realistic training or competitive settings to determine whether music's ergogenic effects are consistent or amplified at higher performance levels, thereby enhancing the external validity of findings.

In addition, there is considerable variability in music intervention protocols, which could influence the observed ergogenic effects. Factors such as music preference, tempo, genre and individual motivation levels vary between studies, making direct comparisons difficult. In team sports, this variability is particularly relevant, as it is difficult to select a single type of music that will suit all players.

## Directions for future research

To further investigate the effects of music on athletic performance and psychophysiological indicators, it is essential to develop studies that address existing limitations and extend current knowledge. Firstly, it is crucial to include international elite athletes in the research to increase the external validity of the results. This will allow the investigation of how music affects performance during high-intensity training and competition, as well as the identification of individual athlete preferences that may be associated with their psychophysiological responses.

Secondly, to ensure reproducibility of results, it is necessary to standardise the parameters of the musical accompaniment. This includes defining optimal tempo ranges (e.g., 120–140 BPM for aerobic activities), controlling volume levels and listening duration, and taking into account the cultural and individual preferences of athletes. Such measures will help to minimise variability and increase data reliability.

Third, to objectively assess the psychological effects of music, it is recommended to use validated tools such as the Profile of Mood States (POMS) to measure mood changes, the Rating of Perceived Exertion (RPE) scale to assess subjective perception of exertion, and motivation questionnaires (e.g., BRUMS). This will provide more accurate data on the effects of music on emotional state and athlete engagement.

In addition, it is important to study the long-term effects of music on performance and psychophysiological indicators. Longitudinal studies will help to understand how the regular use of music in training affects athletes' adaptation, motivation and fatigue levels over months or even years.

Finally, it is necessary to investigate whether the positive effects of music used during warm-up are maintained during competition. This includes analysing the effects of music on cognitive functions such as concentration and decision-making under competitive stress, and comparing the performance of athletes with and without music during warm-up.

## Conclusion

The findings suggest that music can positively impact both physiological and psychological factors, though its application in team settings requires further investigation. Given its safety and accessibility, music may serve as a valuable tool for enhancing performance in various sporting contexts. Future studies should include more detailed information on music usage protocols and involve larger sample sizes, particularly including adult elite athletes.

## Data Availability

The original contributions presented in the study are included in the article/Supplementary Material, further inquiries can be directed to the corresponding author.
